# Acute left side varicocele as primary presentation of renal cell carcinoma

**DOI:** 10.1002/ccr3.6514

**Published:** 2022-11-10

**Authors:** Asaad Moradi, Behnam Shakiba

**Affiliations:** ^1^ Department of urology Firoozgar Hospital, School of Medicine, Iran University of Medical Sciences Tehran Iran

**Keywords:** kidney, neoplasms, renal cell carcinoma, varicocele

## Abstract

A 68‐year‐old man was referred with acute left side varicocele scrotum. Abdominal computed tomography showed a mass lesion in left kidney. The patient underwent radical nephrectomy. Microscopic histopathology confirmed the diagnosis of renal cell carcinoma. The majority of varicocele have a non‐pathological etiology but acute varicocele may indicate retroperitoneal mass.

## Abstract 2

Although the majority of varicocele have a non‐pathological etiology, acute nontraumatic varicocele especially in older patients and tense varicocele in supine position may indicate the presence of a retroperitoneal mass especially renal cell carcinoma.

A 68‐year‐old Iranian man was referred to our clinic with varicose veins had developed in his left hemi scrotum 2 months earlier. He had no history of hematuria or flank pain. On physical examination, an enlarged varicose vein was visible in the left spermatic cord (Figure 1, arrow). Abdominal computed tomography showed an enhancing mass lesion measuring 10 × 12 × 11 cm in left kidney (Figure 2, arrow). With the clinical diagnosis of renal cell carcinoma, patient underwent left side open radical nephrectomy. The operation was uneventful, and the patient was discharged after 4 days with no complications. Microscopic histopathology confirmed the diagnosis of clear renal cell carcinoma. Tumor extends into the renal vein and renal sinus fat (pT3a). Due to lung metastasis, he was referred for sunitinib therapy; unfortunately, he died some months after operation.

**FIGURE 1:**
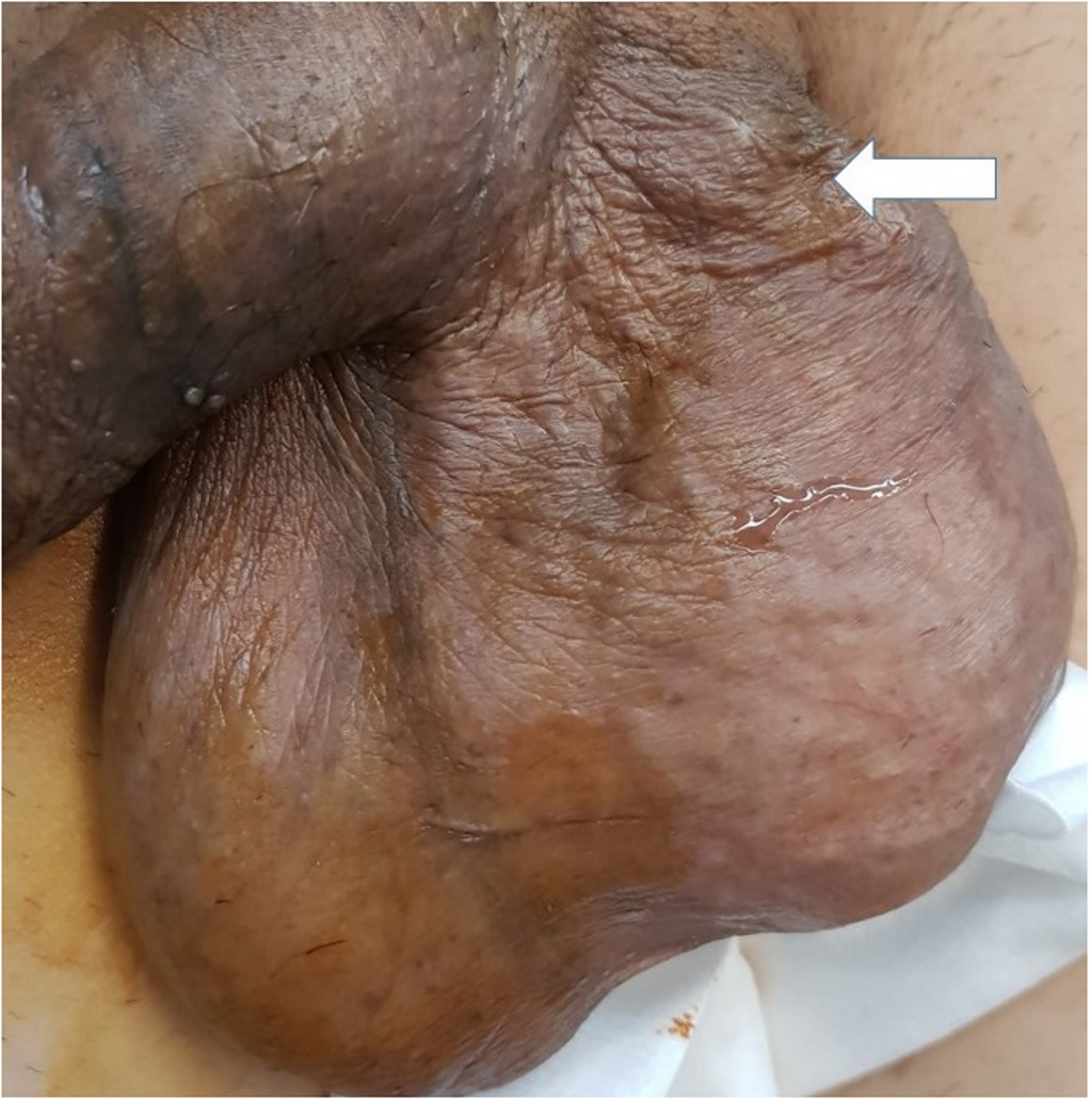
On physical examination, an enlarged varicose vein was visible in the left spermatic cord

**FIGURE 2:**
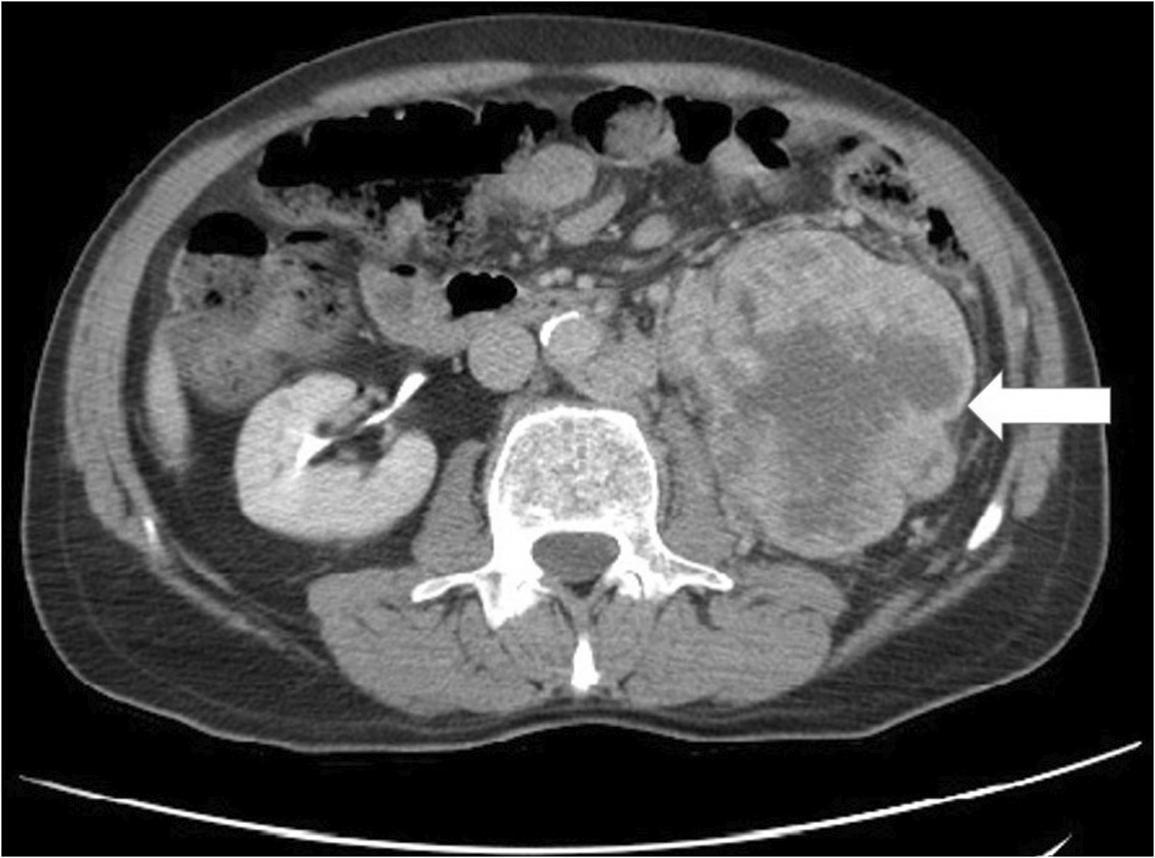
Abdominal computed tomography showed an enhancing mass lesion in left kidney

Varicocele, which is defined as an abnormal venous dilatation and tortuosity of the pampiniform plexus, occurs more commonly on the left side.^1^ Although the majority of varicocele have a non‐pathological etiology, acute nontraumatic varicocele especially in older patients and tense varicocele in supine position may indicate the presence of a retroperitoneal mass especially renal cell carcinoma.^2^ Other causes include retroperitoneal sarcoma, retroperitoneal fibrosis, and lymphoma.^3^

## AUTHOR CONTRIBUTIONS

Behnam Shakiba involved in conceptualization. Asaad Moradi involved in writing–original draft preparation. Behnam Shakiba involved in writing–review and editing. All authors have read and approved the final version of the manuscript.

## CONFLICT OF INTEREST

None.

## ETHICAL APPROVAL

All procedures performed were in accordance with the ethical standards.

## CONSENT

Written informed consent was obtained from the patient to publish this report in accordance with the journal's patient consent policy

## Data Availability

None.
